# Air-Proof Eggs.—Chemical Triumph!

**Published:** 1867-09

**Authors:** 


### Air-Proof Eggs.---Chemical Triumph!

GRANGE G. BECK of 102 Fourth Avenue, New York, near the Cooper Institute, after many years of persistent experiment has perfected a methodby which he can, in ten minutes, at an expense of less than two cents a dozen, place a barrel of fresh eggs in a condition in which they will remain as perfectly fresh as on the day they were laid, for eight months or a year!

The Right is sold to Counties for one thousand dollars, or he will prepare the eggs for two cents a dozen, and will guarantee their remaining fresh.

When it is taken into account that fresh eggs can be purchased in the country, in the early autumn, for ten or fifteen cents a dozen, and are retailed in the large cities three or four months later for four, five, and even six cents apiece, it is clear that a very handsome profit may be made by a person of energy and means, in a very short time. A hundred thousand barrels of fresh eggs can be sold at forty cents a dozen in any winter month, in New York city alone, thus enabling a man to double his money easily within six months.

Egg Dealers, Keepers of Hotels, Boarding-Houses, Restaurants, Groceries and Provision Stores, invited to correspond with the subscriber, GRANGE G. BECK, No. 102, Fourth Avenue, New York.

---

The Congregationalists will send out from Chicago, on the 1st of September, the first number of THE ADVANCE, a religious weekly newspaper of nearly the size of The Weekly Tribune, to be under the editorial charge of the Rev. Wm. Patton, D. D. It is said the capital stock of the ADVANCE Company is $50,000, of which $25,000 has been subscribed in Chicago, and the same amount has been offered, in sums of $5,000 or $10,000, to the other principal Western cities. It is sought, says the CINCINNATI ADVOCATE, by this provision to guard against "a second defection like that of THE INDEPENDENT." James B. T. Marsh, now of THE OBERLIN NEWS, is to be business manager and assistant to the Editor-in-Chief.

---

"TESTIMONIES of American Statesmen and Jurists, to the Truths of Christianity," by Hon. HENRY WILSON, United States Senator from Massachusetts, published by the American Tract Society, 28 Cornhill, Boston. Some of these testimonies, beginning with Washington, are as follows:—

WASHINGTON.—It is impossible to govern the world without God. It is the duty of all nations to acknowledge the Providence of Almighty God, to obey his will, to be grateful for his benefits and humbly implore his protection and favor. I am sure there never was a people who had more reason to acknowledge a divine interposition in their affairs, than those of the United States; and I should be pained to believe that they have forgotten that agency which was so often manifested during the revolution; or that they failed to consider the omnipotence of Him, who is alone able to protect them. He must be worse than an infidel that lacks faith, and more than wicked, that has not gratitude enough to acknowledge his obligations."

JOHN ADAMS.—"The Christian religion, as I understand it, is the brightness of the glory and the express portrait of the eternal self-existing, independent, all powerful and all merciful Creator, Preserver and Father of the Universe; it will last as long as the world. Neither savage nor civilized man, without a revelation could have discovered or invented it. Religion and virtue are the only foundations, not only of republicanism and of all free governments, but of social felicity under all governments and in all the constructions of human society."

THOMAS JEFFERSON.—"I shall need the favor of that Being in whose hands we are, who led our fathers, as Israel of old, from their native land, and planted them in a country flowing with all the necessaries and comforts of life, who has covered our infancy with his providence and our riper years with his wisdom and power, and to whose goodness I ask you to join with me in supplications that he will so enlighten the minds of your servants, guide their counsels and prosper their measures, that whatsoever they do shall result in your good, and shall secure to you the friendship and approbation of all nations."

JAMES MADISON.—"We have been encouraged to feel the guardianship and guidance of that Almighty Being, whose power regulates the doctrines of nations, whose blessings have been so conspicuously displayed to this rising republic, and to whom we are bound to address our devout gratitude for the past, as well as our supplications and best hopes for the future. No people can be bound to adore the invisible hand which conducts the affairs of men, more than the people of the United States. Every step by which they have been advanced to the character of an independent nation seems to have been distinguished by some token of his providential agency."

JAMES MONROE assumed the duties of fourth president of the United States with the expression of a "firm reliance on the protection of Almighty God. Deeply impressed with the blessings which we enjoy, and of which we have such manifold proofs, my mind is irresistably drawn to that Almighty Being, the great source from whence they proceed, and to whom our most grateful acknowledgments are due."

JOHN QUINCY ADAMS.—"So great is my veneration for the Bible, and so strong my belief that when duly read and meditated upon, it is of all books in the world that which contributes to make men good, wise and happy ; that the earlier my children begin to read it, and the more steadily they pursue the practice of reading it throughout their lives, the more lively and confident will be my hopes that they will prove useful citizens to their country, respected members of society, and a real blessing to their parents. I have, myself, for many years, made it a practice to read the Bible through once every year. My custom is to read four or five chapters every morning immediately after rising from bed. It employs about an hour of my time, and seems to me the most suitable manner of beginning the day. You know the difference between right and wrong. You know some of your duties and the obligations you are under of becoming acquainted with them all. It is in the Bible you must learn them, and from the Bible how to practice them."

ANDREW JACKSON.—"It is my fervent prayer to that Almighty being before whom I now stand, and who has kept us in his hands from the infancy of the republic to the present day, that he will so overrule all my intentions and actions, and inspire the hearts of my fellow-citizens, that we may be preserved from dangers of all kind, and continue forever a united and happy people. The Bible is the rock on which our republic rests."

MARTIN VAN BUREN. I only look to the gracious protection of that Divine Being, whose strengthening support I humbly solicit, and whom I fervently pray to look down upon us all. The atonement of Christ is the only remedy and rest for the soul."

GENERAL HARRISON.—"I deem the present occasion sufficiently important and solemn to justify me in expressing to my fellow citizens a profound reverence for the Christian religion, and a thorough conviction that sound morals, religious liberty, and a just sense of religious responsibility are essentially connected with all true and lasting happiness. I think I enjoy religion and delight in the duties of a child of God, and have concluded to unite with the Church of God, as soon as my health will permit me to go out."

ABRAHAM LINCOLN.—"To-day I leave you. I go to assume a task more difficult than that which devolved on General Washington. Unless the great God who assisted him, shall be with and aid me, I cannot prevail, but if the same omniscient mind and the same almighty arm that directed and protected him shall guide and support me, I shall not fail, I shall succeed. Let us pray that the God of our fathers may not forsake us now. To him I commend you all. Permit me to ask, that with equal sincerity and faith, you will all invoke his wisdom and guidance for me.

BENJAMIN FRANKLIN.—"I have lived a long time, and the longer I live, the more convincing proofs I see of this truth, that God governs in the affairs of men. And if a sparrow cannot fall to the ground without His notice, is it probable that an empire can rise without his aid? We have been assured in the sacred writings, that except the Lord build the house, they labor in vain who build it; I therefore move that henceforth prayers imploring the assistance of Heaven, and its blessings on our deliberations, be held in Congress every morning, before we proceed to business."

